# Influence of the microwave technology on solid dispersions of mefenamic acid and flufenamic acid

**DOI:** 10.1371/journal.pone.0182011

**Published:** 2017-07-31

**Authors:** Sultan Alshehri, Faiyaz Shakeel, Mohamed Ibrahim, Ehab Elzayat, Mohammad Altamimi, Gamal Shazly, Kazi Mohsin, Musaed Alkholief, Bader Alsulays, Abdullah Alshetaili, Abdulaziz Alshahrani, Bander Almalki, Fars Alanazi

**Affiliations:** 1 Department of Pharmaceutics, College of Pharmacy, King Saud University, Riyadh, Saudi Arabia; 2 Department of Pharmaceutics, Faculty of Pharmacy, Assiut University, Assiut, Egypt; 3 Department of Pharmaceutics, College of Pharmacy, Prince Sattam Bin Abdulaziz University, Al-Kharj, Saudi Arabia; Waseda University, JAPAN

## Abstract

The present studies were undertaken to develop solvent-free solid dispersions (SDs) for poorly soluble anti-inflammatory drugs mefenamic acid (MA) and flufenamic acid (FFA) in order to enhance their *in vitro* dissolution rate and *in vivo* anti-inflammatory effects. The SDs of MA and FFA were prepared using microwaves irradiation (MW) technique. Different carriers such as Pluronic F127^®^ (PL), Eudragit EPO^®^ (EPO), polyethylene glycol 4000 (PEG 4000) and Gelucire 50/13 (GLU) were used for the preparation of SDs. Prepared MW irradiated SDs were characterized physicochemically using differential scanning calorimetry (DSC), thermogravimetric analysis (TGA), Fourier transform infra-red (FT-IR) spectroscopy, powder X-ray diffraction (PXRD) and scanning electron microscopy (SEM). The physicochemical characteristics and drug release profile of SDs were compared with pure drugs. The results of DSC, TGA, FT-IR, PXRD and SEM showed that SDs were successfully prepared. *In vitro* dissolution rate of MA and FFA was remarkably enhanced by SDs in comparison with pure MA and FFA. The SDs of MA and FFA prepared using PEG 400 showed higher drug release profile in comparison with those prepared using PL, EPO or GLU. The dissolution efficiency for MA-PEG SD and FFA-PEG SD was obtained as 61.40 and 59.18%, respectively. Optimized SDs were also evaluated for *in vivo* anti-inflammatory effects in male Wistar rats. The results showed significant % inhibition by MA-PEG (87.74% after 4 h) and FFA-PEG SDs (81.76% after 4 h) in comparison with pure MA (68.09% after 4 h) and pure FFA (55.27% after 4 h) (P<0.05). These results suggested that MW irradiated SDs of MA and FFA could be successfully used for the enhancement of *in vitro* dissolution rate and *in vivo* therapeutic efficacy of both drugs.

## Introduction

Solubility of drug molecules affects the *in vitro* dissolution rate and consequently *in vivo* absorption and bioavailability of the drugs. Thus, the drug solubility plays an important role in the therapeutic achievement of a drug. Moreover, the preferred drug concentration in systemic circulation and the pharmacological response depend on the drug solubility [1, 2]. The number of limitations such as need of high doses, increase administration frequencies and increase side effects may result from poorly soluble drugs [3].

The majority of the recently formulated drugs exhibit low bioavailability due to their poor aqueous solubility and dissolution rate [4]. The dissolution rate of poorly soluble drugs in the gastrointestinal (GI) fluids is the rate-limiting step for the absorption of drugs in systemic circulation. Thus, the solubility and dissolution rate of such drugs must be enhanced in order to enhance their bioavailability [5]. Improving the solubility of poorly soluble drugs leads to enhancement in the dissolution rate and drug release and consequently drug bioavailability [6]. Different techniques have been investigated in order to improve the solubility of poorly soluble drugs e.g. solubilization in surfactant system [7, 8], complexation [8], micronization [1], drug derivatization [9], solid dispersion [1–6], cosolvent technique [10], nanoparticles [11], nanoemulsions/self-nanoemulsifying drug delivery systems [12–14] and co-crystal technology etc. [15].

Microwave (MW) irradiation is relatively a novel method used for solubility and bioavailability enhancement of thermo-stable and drying materials [16, 17]. MW equipment depends on the use of the electromagnetic waves between the radio and infrared frequencies over the range of 0.3–300 GHz. These waves migrate within the materials, causing oscillation of the molecules and finally generating heat [18]. MW technique is different in comparison with conventional heating in which the surface of the material heats first and then the heat moves inward. In MW technique, the heat is generated inside the material and then passes to the entire volume with the constant heating rate [19]. MWs have the ability to penetrate any material leading to heat production everywhere in the material at the same time. This is due to the absorption of MW energy by the dipolar moment of the molecules converting it into the heat. There are several advantages of MW technology in comparison with other techniques such as energy saving, non-contact heating, quick start-up and stopping, low operating cost, portability of equipment and processes, the ability to treat waste in-situ, rapid volumetric heating and no overheating at the surface [20]. MW energy converts the crystalline form of the drug to the amorphous forms and hence improves drug dissolution rate which could results in enhanced drug bioavailability [16].

**Fig 1:**
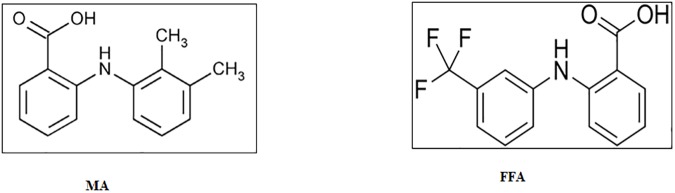
Molecular structures of MA and FFA.

Mefenamic acid (MA) and flufenamic acid (FFA) are non-steroidal anti-inflammatory drugs (NSAIDs) that are *N*-phenylanthranilic acid derivatives [21]. The molecular structures of MA and FFA are presented in Fig 1. These NSAIDs are potent analgesic, antipyretic and anti-inflammatory drugs which are being applied in the treatment of rheumatoid arthritis, osteoarthritis and other painful musculosketal conditions [21–23]. They have the capacity to inhibit cyclooxygenase and may antagonize the certain effects of prostaglandins [24]. They belong to class II category under biopharmaceutical classification system (BCS) i.e., they exhibit low aqueous solubility and high permeability [25]. Thus their solubility has to be increased to enhance their dissolution rate and consequently to improve their bioavailability. The SDs of MA and FFA have been prepared and evaluated using different techniques and different carriers in order to enhance their solubility, dissolution and bioavailability [26–31]. However, the SDs of these drugs have not been investigated using a solvent-free MW technology in literature. Therefore, the objective of the present work was to enhance the dissolution rate and anti-inflammatory effects of MA and FFA via MW technique using four different carriers including Pluronic F127^®^ (PL), Eudragit EPO^®^ (EPO), polyethylene glycol 4000 (PEG 4000) and Gelucire 50/13 (GLU).

## Materials and methods

### Materials

MA (purity 99.3%), FFA (purity 99.5%) and PL (purity 99.6%) were obtained from “Sigma Aldrich (St. Louis, MO)”. GLU (purity 99.2%) was obtained as a kind gift sample from “Gattefosse (Lyon, France)”. EPO (purity 99.1%) was obtained as a kind gift sample from “Evonik (Darmstadt, Germany)”. PEG 4000 (purity 99.0%) was obtained from “E-Merck (Darmstadt, Germany)”. All other chemicals used were of high purity and analytical grade.

### Preparation of SDs

In this particular study, different SDs of MA and FFA were prepared using domestic MW irradiation (Samsung Model ME0113M1) [16, 17]. Each pure drug was blended with the GLU, EPO, PEG or PL at fixed drug carrier ratio of 1:5 w/w and sieved using mesh size no. 45 to obtain uniform particle size and to prevent powder coagulation. Briefly, a precise amount of the drug and the carrier (approximately 1 g) were gently mixed for about 5 min. The MW power was set at 900 W. The samples of interest were placed in a glass beaker and subjected to MW irradiation one by one at the exact place. The time was calculated depending upon the melted sample and obtained a homogenous mass to form SD system (Table 1). The samples were allowed to cooled and solidified and placed in the desiccator for about 48 h to remove any residual moisture [16]. Then, the samples were pulverized using porcelain mortar and pestle for 10 min and sieved using sieved mesh size no. 45 to obtain uniform particle size [17]. The samples were kept in the refrigerator in glass bottles till further use.

**Table 1:** MW conditions used in the preparation of SDs of MA and FFA prepared by MW irradiation using different carriers.

	MW time (min)			
Carriers	PL	EPO	PEG 4000	GLU
MA	7	23	7	6
FL	4	20	6	3
Dug carrier ratio	1: 5			
Power level (W)	900			

### Thermal gravimetric analysis (TGA)

TGA analysis was performed using “Pyris 1 TGA (Perkin Elmer, Waltham, MA)” apparatus outfitted with auto sampler to analyze the thermal stabilities of pure chemicals (MA, FFA, GLU, EPO, PL and PEG). The experiments were carried out in the heating range of 25–250°C with accelerated rate of 10 C/min. Each sample weighed approximately 3.0 mg and analyzed under a nitrogen purge of 20 ml/min. The TGA results were evaluated according to the weight loss of the treated samples subtracted from the original one.

### Differential scanning calorimetry (DSC)

DSC analysis of pure MA, FFA and eight different formulations were determined using “DSC apparatus (DSC-8000, Perkin Elmer, Waltham, MA)” over the temperature range of 25.00°C to 250.00°C at 10.00°C/min and recorded under a nitrogen purge of 20 ml/min. This instrument was equipped with auto sampler and chiller. The samples of interest were accurately weighed (around 3.0 mg) and placed in an aluminium pan and hermetically sealed. This instrument was equipped with Pyris manager software “(Perkin Elmer, Waltham, MA)” to evaluate the solid state characterization and thermal stability of each sample.

### Fourier transform infrared spectroscopy (FTIR)

The FTIR spectra of pure MA, FFA, carriers and their MW SDs were obtained using a “Perkin Elmer FTIR spectrum BX (Perkin Elmer, Marlborough, MA)”. The materials were prepared as KBr pellets and spectra were collected in the range of 4400 to 350 cm^-1^ wavenumber using 3 scans and 2 cm^-1^ resolution [32, 33]. The data were analyzed using IR solution software (version 1.10, Marlborough, MA).

### Scanning electron microscope (SEM)

The changes in the shape and surface morphology of the pure drugs and SD samples were evaluated using SEM. Photomicrographs of the samples were taken using SEM microscope (Zeiss EVO LS10; Cambridge, UK). The samples were fixed on stubs using both side adhesive carbon tape (SPI Supplies, West Chester, PA) and coated under vacuum with gold in a Q150R sputter coater unit from Quorum Technologies Ltd. (East Sussex, UK) in an argon atmosphere at 20 mA for 60 sec.

### Powder X-Ray diffraction (PXRD)

PXRD of pure drugs and SD samples were evaluated by Ultima IV diffractometer (Rigaku Inc. Tokyo, Japan at College of Pharmacy, King Saud University, KSA) over the 3−60° 2θ range at a scan speed of 1°/min. The tube anode was Cu with Ka = 0.1540562 nm mono chromatized with a graphite crystal (Rigaku Inc. Tokyo, Japan). The pattern was collected at 40 kV of tube voltage and 40 mA of tube current in step scan mode (step size 0.02°, counting time 1 second per step). This study was set to determine the crystallinity of the pure drugs in the SD system.

### UPLC-UV analysis for MA and FFA

The analysis of MA and FFA was performed using a validated ultra performance liquid chromatography-ultra-violet (UPLC-UV) method. The amount of MA and FFA was determined by injecting samples into a UPLC (Acquity^®^ UPLC, Waters Inc., Bedford, MA) with UV detection at 278 nm (for MA) and 286 nm (for FFA). Briefly, separation employed reverse-phase isocratic elution using a mobile phase consisting of a mixture of 0.05 M ammonium acetate buffer (adjusted to pH = 2 with phosphoric acid) and acetonitrile (65:35% v/v) run at flow rate of 0.3 ml/min and injection volume was 1 μl. The PDA detector was set to acquire 3D data from 210 to 400 nm while the 2D channel was recorded at 336 nm. The column (Acquity^®^ UPLC BEH C_18_ column, 2.1× 50 mm, 1.7 μm, Waters, Bedford, MA) was used and temperature was set at 40°C. The linearity was excellent with R^2 =^ 0.999 and the peak was resolved at a retention time of 1.02 min (for FFA) and 1.3 min (for MA). This method was employed in determination of the drug content in dissolution samples.

### *In vitro* dissolution studies

*In vitro* dissolution tests were carried out using USP 24 dissolution rate test apparatus Type II (paddle type, rotating at 100 rpm) using an automated dissolution tester (LOGAN Instrument Corp., Somerset, NJ). The SDs were filled in hard gelatin capsules which were placed in a dissolution medium of 900 ml 0.05 M phosphate buffer (pH = 7.4) for 2 h maintained at 37°C. The samples of 5 ml each were withdrawn at predetermined time intervals and replaced with freshly prepared dissolution medium. The samples were filtered and analyzed by injection into UPLC as mentioned above in the previous section. The mean value of the percentage of drug release was plotted against the time interval.

### *In vivo* anti-inflammatory studies

Based on physicochemical characterization and maximum drug release profiles, SDs MA/PEG and FFA/PEG were selected for *in vivo* anti-inflammatory studies. Therefore, *in vivo* anti-inflammatory studies were performed on SDs MA/PEG and FFA/PEG in order to compare their anti-inflammatory effects with pure MA and pure FFA. Thirty male Wistar rats (weighing from 200–250 g) were obtained from “Animal Care and Use Center of College of Pharmacy at King Saud University, Riyadh, Saudi Arabia”. Approval to carry out *in vivo* anti-inflammatory studies was obtained from Animal Ethics Committee of King Saud University, Riyadh Saudi Arabia. Institutional guidelines were strictly followed in order to perform these studies. All the animals were given standard laboratory conditions of “temperature and relative humidity”. The rats were kept in plastic cages and provided free access to standard laboratory pellet diet and water *ad libitum*. The anti-inflammatory effects were investigated by the Carrageenan-induced hind paw edema method developed by Winter et al. 1965 [34]. The rats were randomly divided into 5 groups with 6 rats in each group. Group I rats were administered Carrageenan only and served as control group. Group II and III rats were administered MA and FFA suspension, respectively. Group IV and V rats were administered MA/PEG and FFA/PEG SDs. MA suspension (10 mg/kg), FFA suspension (10 mg/kg), MA/PEG SD (containing 10 mg/kg of MA) and FFA/PEG SD (containing 10 mg/kg of FFA) were administered orally half an hour before subplantar injection of Carrageenan in the right paw. Paw edema was induced by injecting 0.1 ml of a 1% w/v suspension of Carrageenan in ultra-pure water. The initial and final paw volume of each rat was measured at regular interval of time (1, 2, 3, 4, 5 and 6 h) after injection using a digital Plethysmometer (Ugo Basile, Italy). Percent inhibition of edema produced by each formulation-treated group was calculated with respect to control group using its standard formula reported in literature [34, 35].

### Statistical analysis

The results of this study were analyzed statistically using one way analysis of variance (ANOVA) analysis followed by Dennett’s test using GraphpadInstat software (San Diego, CA). The P vale of <0.05 was considered as statistically significant.

## Results and discussion

### MW process

Before starting the MW process, the study of the drug-carrier physicochemical characteristics is important which would help to adjust and optimize the experiment parameters that will lead to save time and efforts. The selection of suitable excipients is highly recommended in order to obtain excellent results and perfect products. In this study, four different carriers were selected depending upon their melting point, elasticity and their ability to convert the crystalline form of drug to an amorphous form. The obtained physical mixtures (PM) were subjected to MW irradiation at 900 W at different time of interval. PM powders were fine powder and easy to handle. When the binary mixtures of drugs (MA or FFA) with different carriers such as PL, EPO, GLU and PEG were prepared and subjected to MW, fine powder (mill and sieve) were obtained except for one mixture i.e. FFA/GLU. It was because of the low melting point of the drug and carrier. This mixture was also sticky and difficult to handle. Therefore, the binary mixture of this drug with the carrier was placed at -80°C for about 1 h in order to allow solidification. The solidified mixture was then subjected to grinding and sieving in order to obtain powder with uniform size. MW conditions used in the preparation of SDs of MA and FFA with suitable carriers are presented in Table 1.

### TGA analysis

**Fig 2:**
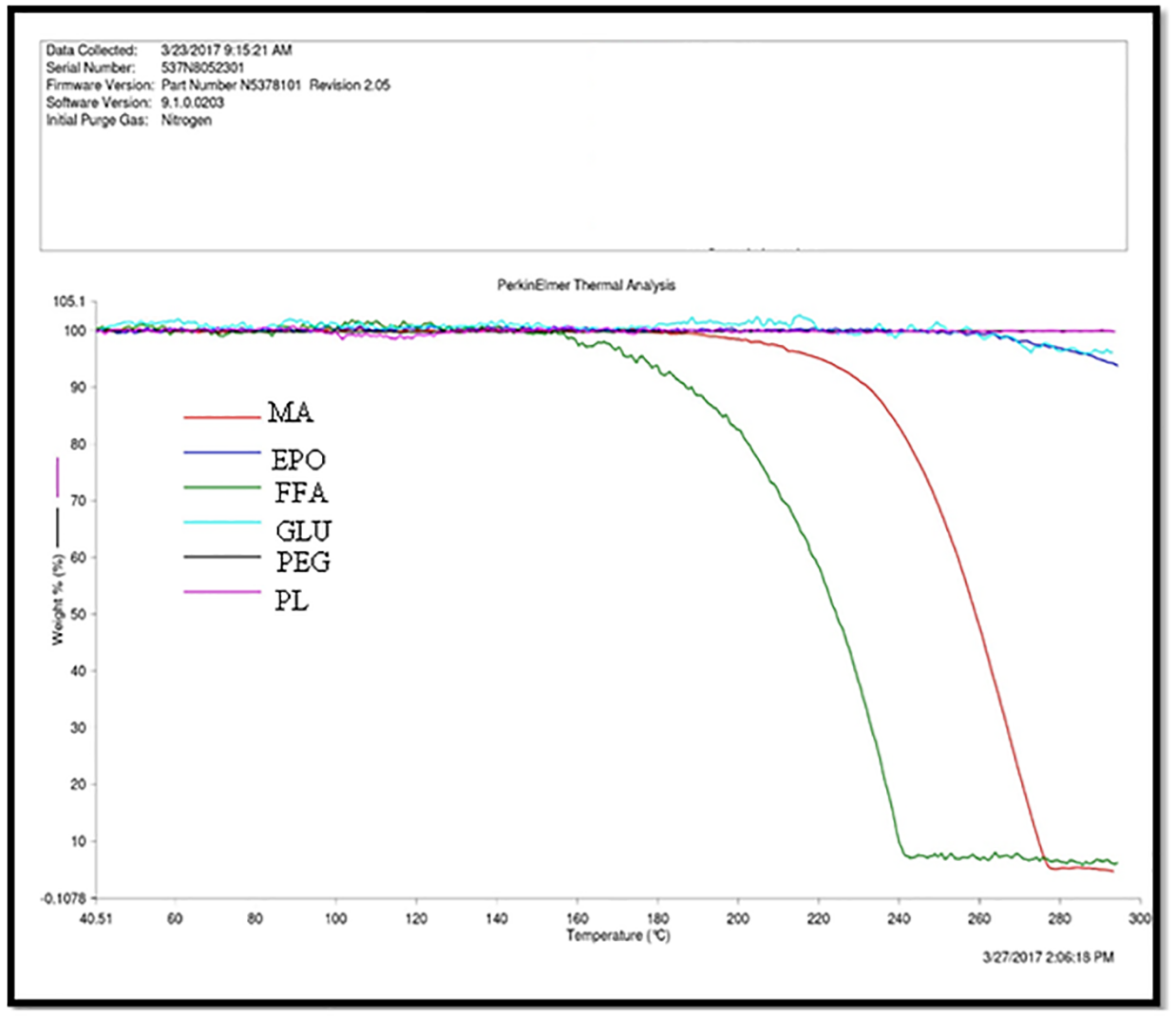
TGA spectra of pure MA, pure FFA and different carriers.

TGA spectra of pure MA, pure FFA and different carriers are presented in Fig 2. TGA spectra of pure MA presented weight loss at around 238.21°C (Fig 2). This weight loss was possible due to decomposition of MA at around 238.21°C. The results of TGA analysis showed that MA does not exist in polymorphic form. The weight loss of MA due to decomposition was obtained as 0.92% (w/w). However, the TGA spectra of pure FFA presented weight loss at around 191.77°C (Fig 2). This weight loss was possible due to decomposition of FFA at around 191.77°C. The results of TGA analysis of FFA also showed that FFA does not exist in polymorphic form. The weight loss of FFA due to decomposition was obtained as 0.13% (w/w). On the other hand, the TGA spectra of different carriers showed no decomposition of PL, GLU, EPO and PEG, indicating that these carriers did not exist in crystalline form.

### DSC analysis

**Fig 3:**
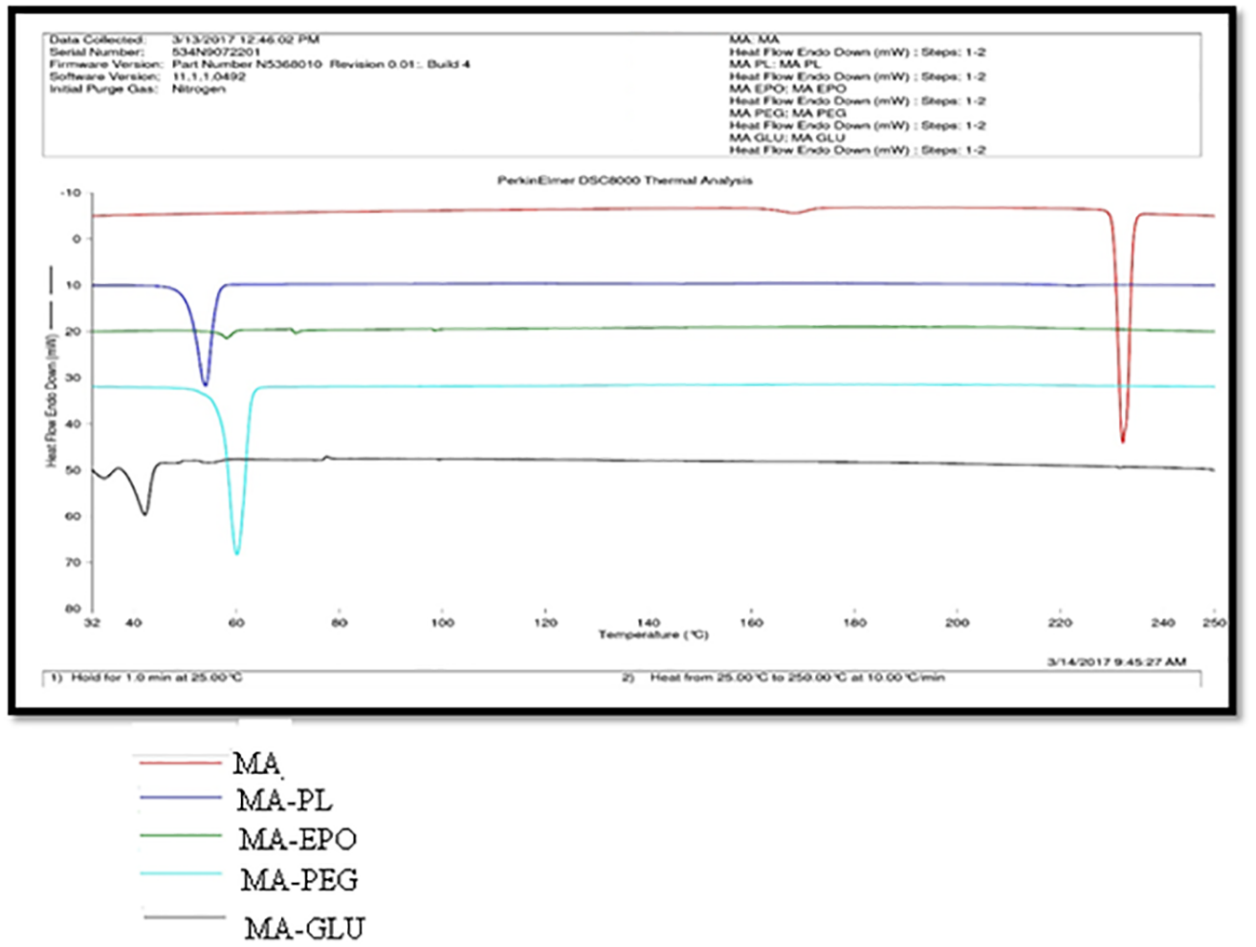
DSC spectra of pure MA and different SDs.

**Fig 4:**
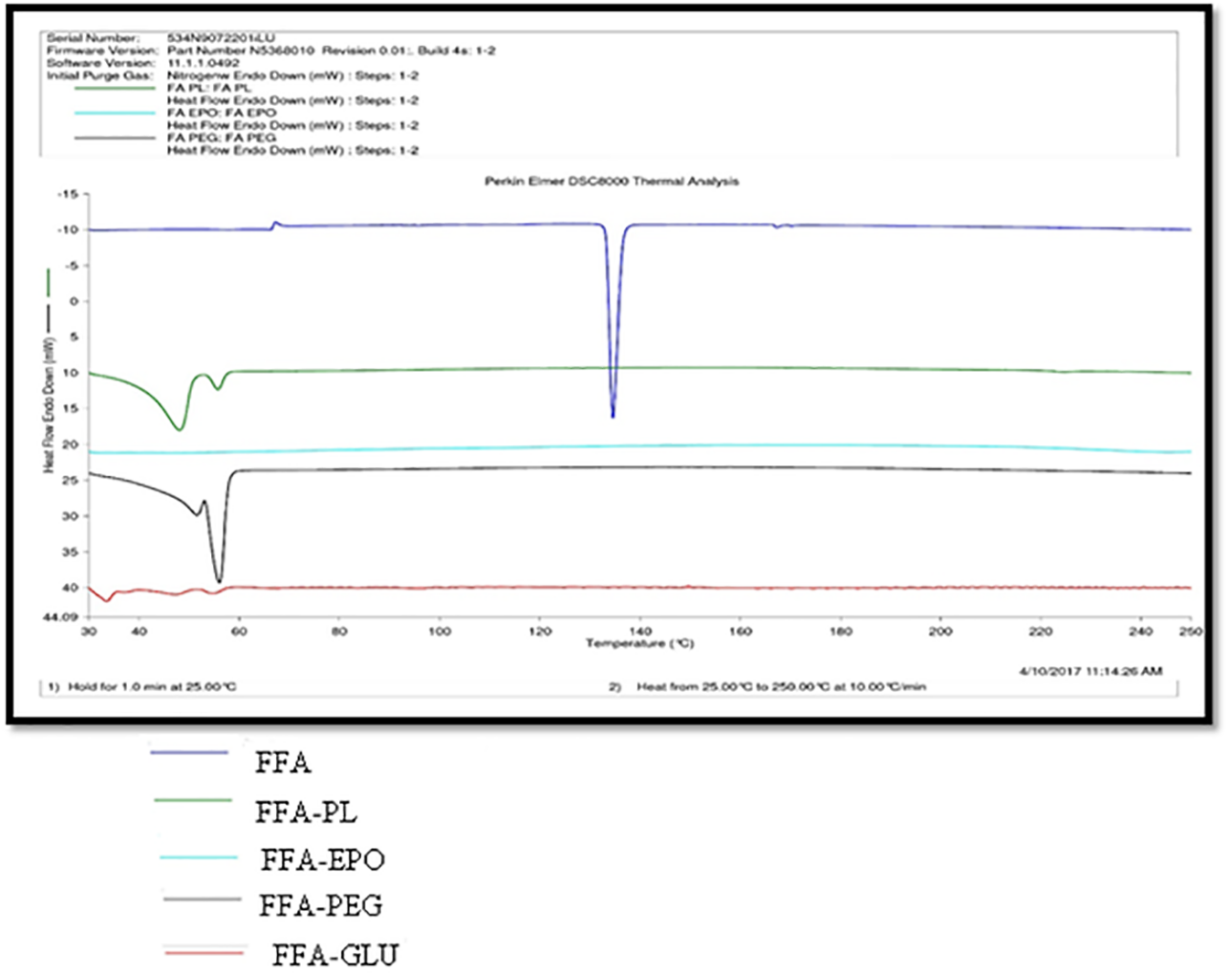
DSC spectra of pure FFA and different SDs.

DSC analysis was performed to evaluate drug–carrier interactions in different SDs [22, 23]. DSC thermograms of pure MA and its SDs are presented in Fig 3. DSC thermogram of pure MA showed a sharp and crystalline endothermic peak at fusion temperature of 232.52°C with fusion enthalpy of 37.63 kJ/mol. However, the DSC thermograms of SDs prepared using different carriers showed a very poor peak. The crystalline peak of MA was either disappeared or shifted to lower temperatures in case of different SDs. This observation indicated the amorphization of MA into SDs. Amorphization of MA was possible due to molecular interactions of MA with different carriers. The fusion temperature of MA has been reported as 232.11°C in literature [36]. The fusion temperature of MA was obtained as 232.52°C in this work. This value was much closed with literature value. These results indicated good agreement of DSC results with literature data of MA [36]. The DSC thermograms of pure FFA and its SDs are presented in Fig 4. The DSC thermogram of pure FFA showed a sharp and crystalline endothermic peak at fusion temperature of 134.57°C with fusion enthalpy of 29.80 kJ/mol. However, the DSC thermograms of SDs prepared using different carriers showed a very poor peak. The crystalline peak of FFA was either disappeared or shifted to lower temperatures in case of different SDs. This observation indicated the amorphization of FFA into SDs. Amorphization of FFA was possible due to molecular interactions of FFA with different carriers. The fusion temperature of FFA has been reported as 132.10°C in literature [37]. The fusion temperature of FFA was obtained as 134.57°C in this work. This value was much closed with literature value of FFA. These results indicated good agreement of DSC results with literature data of FFA [37]. The single and sharp crystalline peaks of pure MA and pure FFA indicated that both drugs were in crystalline form and did not show any evidence of polymorphic transformations. Overall, the results of DSC spectra indicated that SDs of MA and FFA were successfully prepared by MW technology.

### FTIR spectral analysis

**Fig 5:**
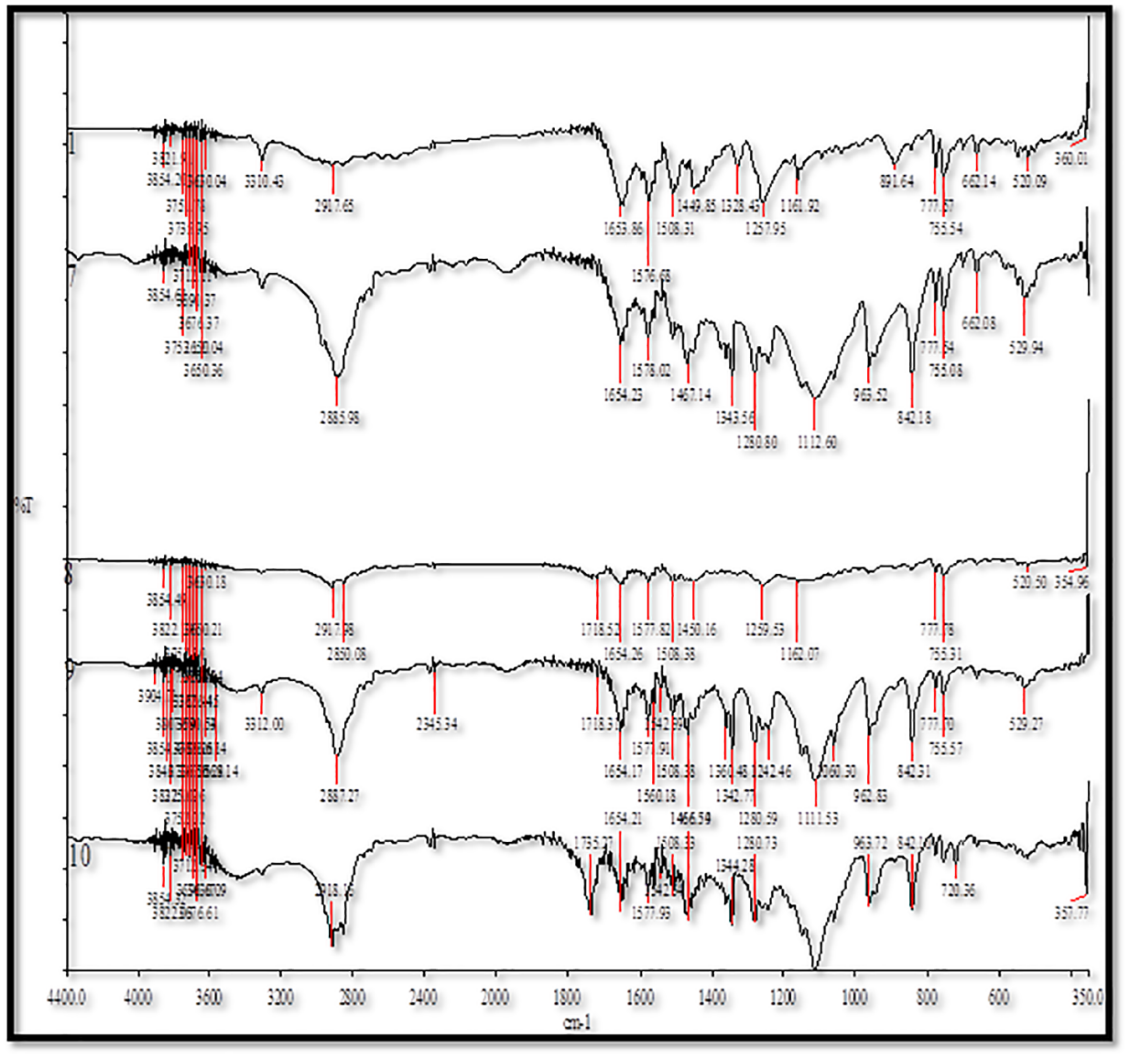
FTIR spectra for 1. pure MA, 7. MA-PL, 8. MA-EPO, 9. MA-PEG and 10. MA-GLU.

The FTIR spectra of pure MA and its MW SDs are presented in Fig 5. The IR energy is sufficient to stretch or bend the bonds of a particular molecule. Therefore, different chemical substances have a specific IR spectrum which serves as a fingerprint. When the molecules are dispersed in polymers, the newly formed bonds are detected using these methods [38–40].

For PL, the FTIR spectrum of C-H stretching showed a distinct peak at around 2889 cm^−1^. The C-O-C vibrational stretching is assigned to the peak of 1112 cm^−1^. EPO had a vibrational stretching of C = O at 1734 cm^−1^. Vibrational stretching of O-H and C-O-C for PEG 4000 is clear at 3447 and 1112 cm^−1^, respectively. The characteristic peaks found at 1112, 1736, 2888, and 3448 cm^−1^ is assigned to C-O, C = O, C-H, and O-H, respectively [29, 41–43].

The probability is high for a hydrogen bonding in the amine and the carbonyl groups for MA. The FTIR spectrum for N-H, C = O and C = C is attributed to 3310, 1654 and 1576 cm^−1^ wavenumbers. For the MW formulation between MA and PL, all the assigned peak showed no significant difference in comparison with pure drugs except for the ether vibrational which shifted to higher wave number of 2886 cm^−1^, indicating a probability of a hydrogen bonding with C = C in the MA. No clear shifts in the vibrational bands were found in the MA with either EPO, or PEG 4000 or GLU mixtures (Fig 5).

**Fig 6:**
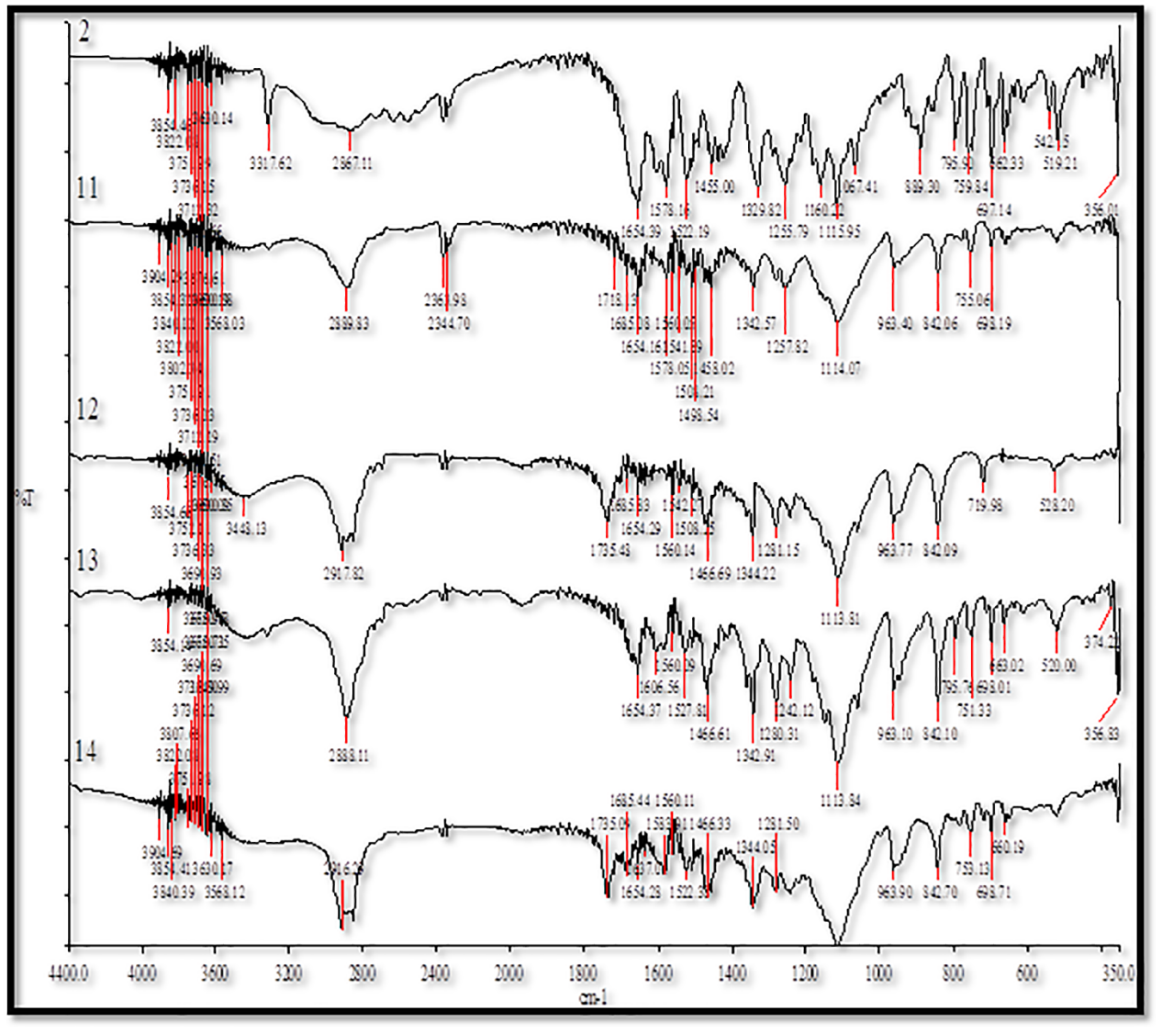
FTIR spectra for 2. FFA, 11. FFA-PL, 12. FFA-EPO, 13. FFA-PEG and 14. FFA-GLU.

The FTIR spectra of pure FFA and its MW SDs are presented in Fig 6. FFA showed a characteristic peak due to the stretching of secondary amine N-H at 3318 cm^−1^. The C = O and C = C vibrational wavenumbers are clear at 1654 cm^−1^ and 1578 cm^−1^, respectively [44, 45]. No significant shift in the peaks between FFA and PL were recorded. For FFA-EPO, a minor shift in carbonyl functional group at 1735 cm^−1^ was recorded which might indicate a hydrogen boding in the mixture. The shift of the vibrational peaks at 2888 and 1114 cm^−1^ indicated hydrogen bonding between ether group in PEG 4000 and methyl group in FFA. No shift in the vibrational bands was found in the FFA-GLU.

The disappearance of the N-H group in different mixtures is ascribed to the disruption of bonding between the FFA or MA molecules and forming new bonds with the carriers.

### SEM analysis

**Fig 7:**
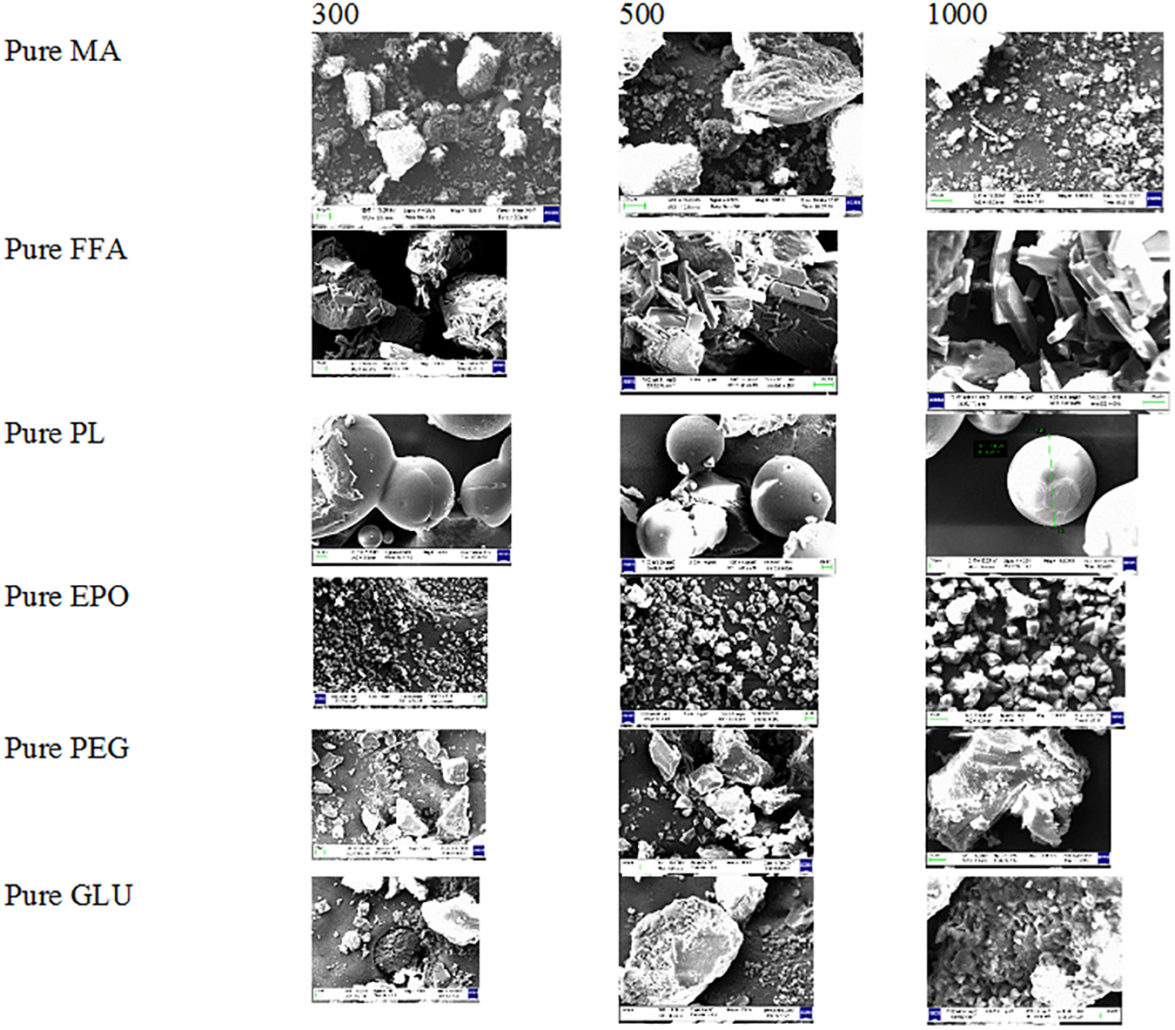
SEM images of pure MA, pure FFA and different carriers.

**Fig 8:**
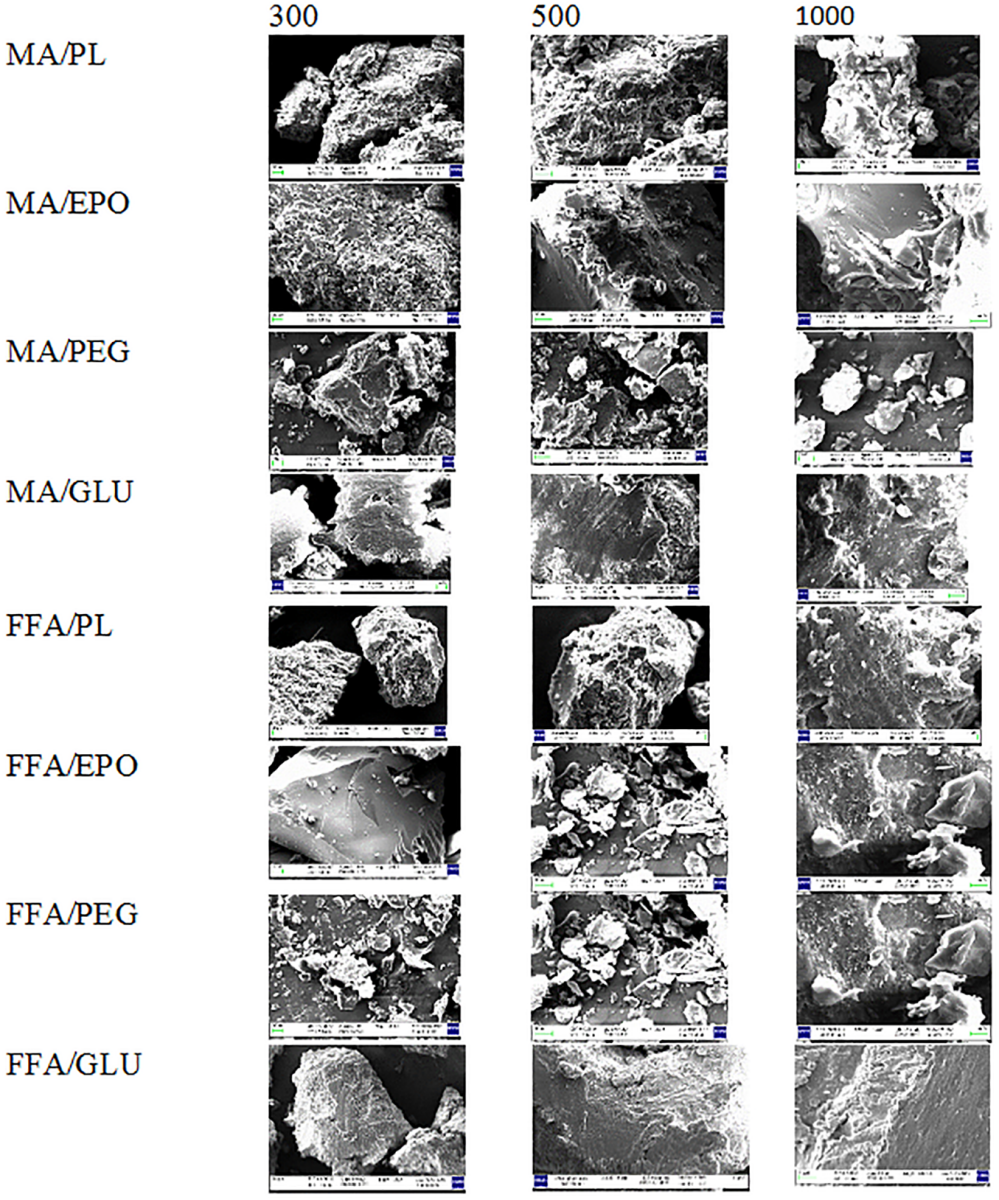
SEM images of different SDs of MA and FFA.

The SEM images of pure MA, pure FFA, pure carriers and SDs of MA and FFA are presented in Figs 7 and 8. The SEM images of pure MA and pure FFA showed clear crystals of these drugs with larger particle size (Fig 7). However, the SEM images of different carriers showed amorphous nature of these carriers (Fig 7). When, MA and FFA were converted into SDs, their morphology changed to amorphous states with fine particle size (Fig 8). Overall, the SEM images showed that SDs of MA and FFA were in amorphous states.

### PXRD spectral analysis

**Fig 9:**
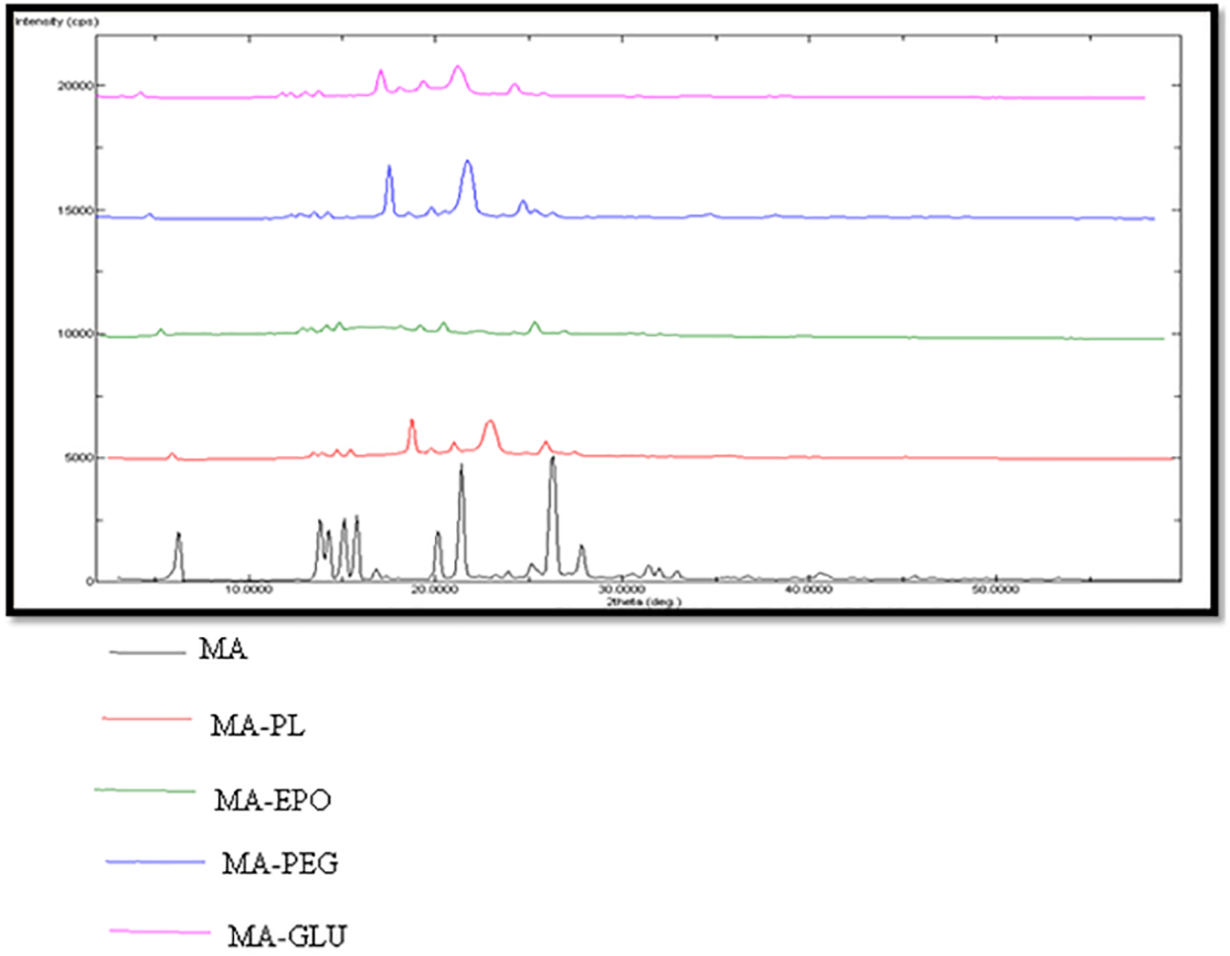
PXRD spectra of pure MA and different SDs.

**Fig 10:**
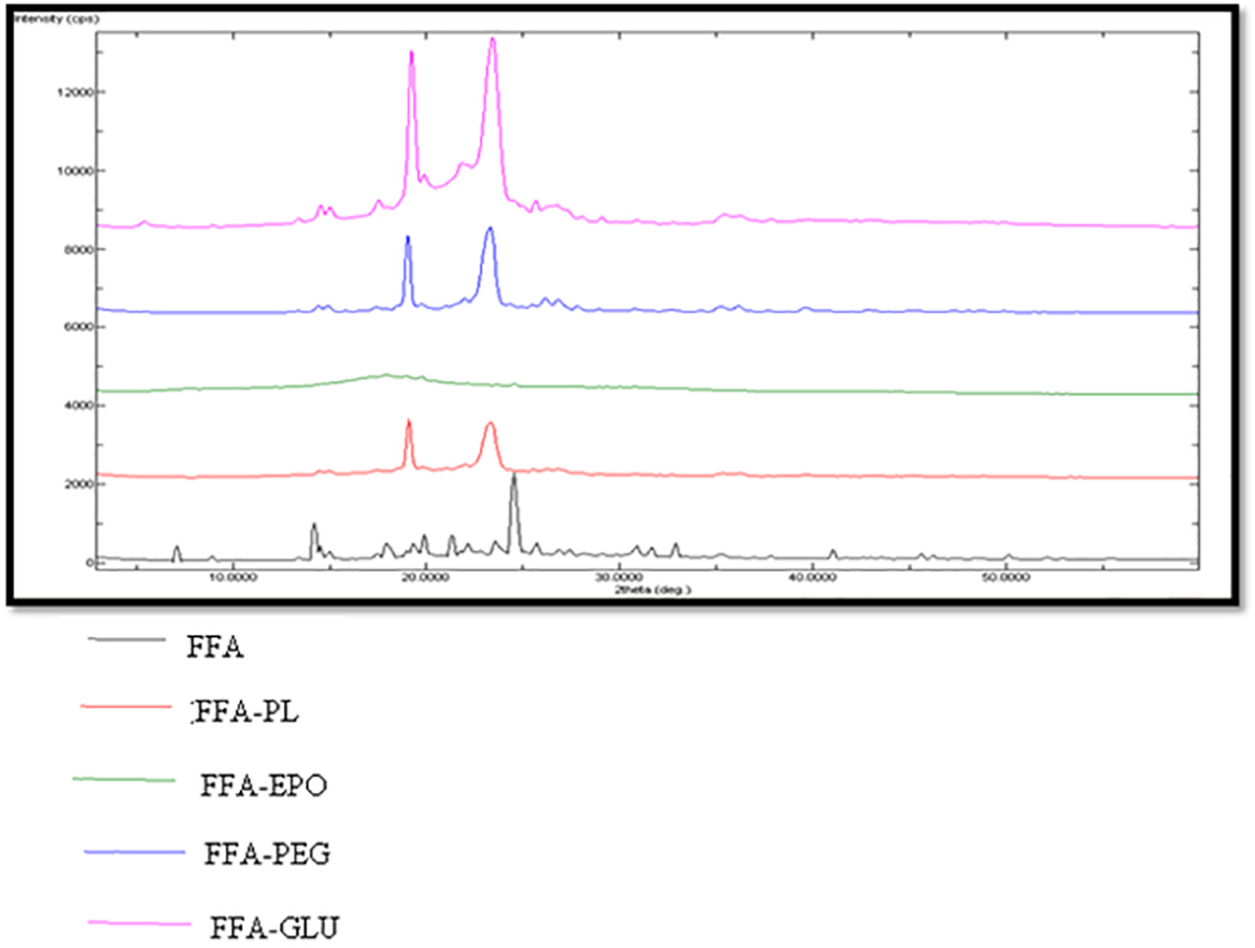
PXRD spectra of pure FFA and different SDs.

The PXRD spectra were recorded in order to evaluate crystalline and amorphous nature of different MW SDs. The PXRD spectra of pure MA and its MW SDs are presented in Fig 9. The PXRD spectra of pure MA showed characteristics sharp crystalline peaks at different 2 θ values due to crystalline nature of MA. However, the PXRD spectra of SDs prepared using different carriers showed either disappearance or shifting of characteristics peaks of MA. This observation again indicated the amorphization of MA into SDs. Amorphization of MA was possible due to molecular interactions of MA with different carriers. The PXRD spectra of pure FFA and its MW SDs are presented in Fig 10. The PXRD spectra of pure FFA also showed characteristics sharp crystalline peaks at different 2 θ values due to its crystalline nature. However, the PXRD spectra of SDs prepared using different carriers showed either disappearance or shifting of characteristics peaks of FFA. This observation again indicated the amorphization of FFA into SDs. Amorphization of FFA was possible due to molecular interactions of FFA with different carriers.

### *In vitro* dissolution studies

**Fig 11:**
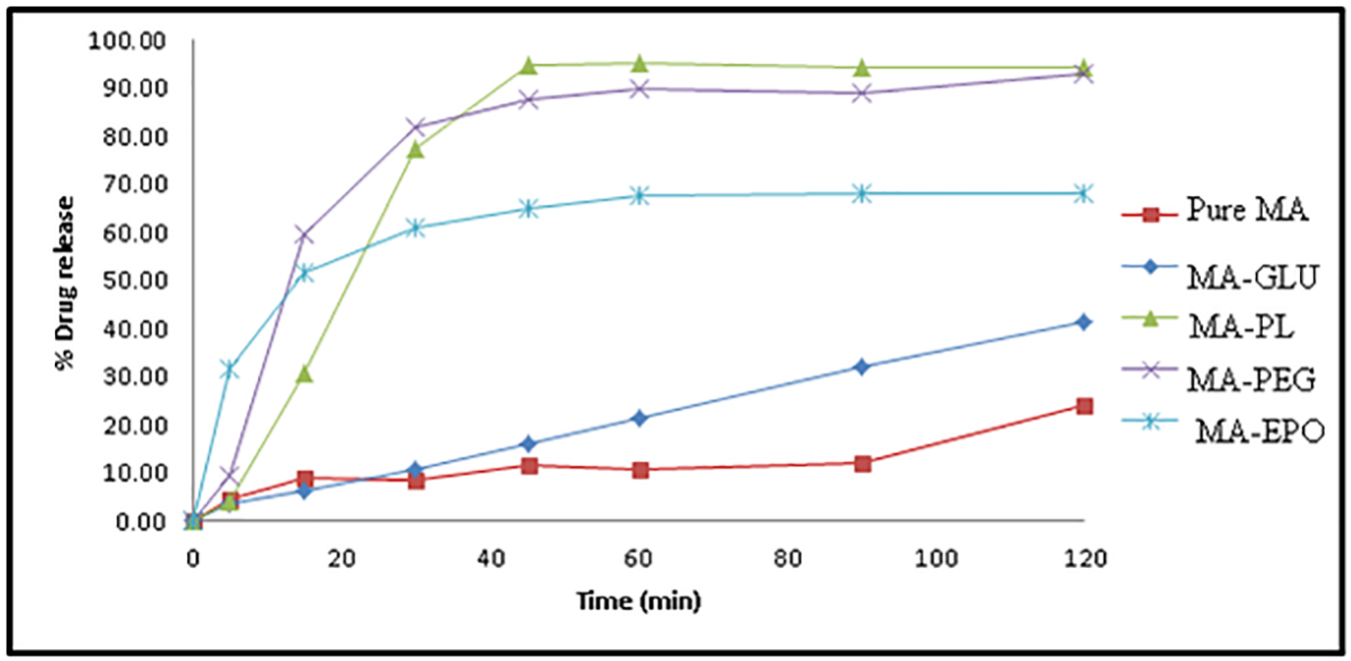
*In vitro* drug release profile of MA from pure MA and its SDs.

**Fig 12:**
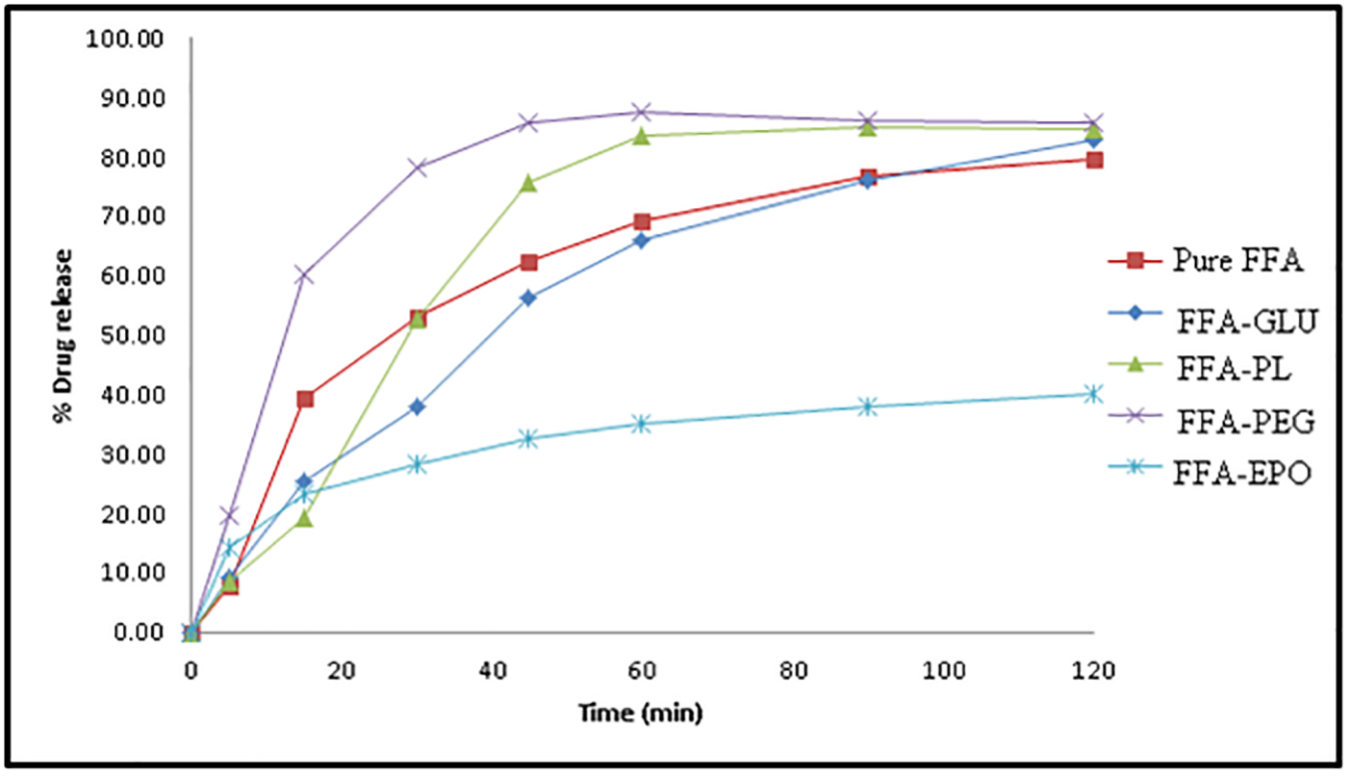
*In vitro* drug release profile of FFA from pure FFA and its SDs.

The *in vitro* dissolution profiles of MA and FFA from their binary systems in different polymers at a drug: polymer ratio of 1: 5 (prepared by MW SD) are displayed in Figs 11 and 12, respectively. Pure MA showed a very poor dissolution rate (Fig 11) in which only 4.63% of drug was released initially after 5 min with DE% of 7.51% after 60 min (Table 2). Similarly, pure FFA exhibited a poor dissolution (Fig 12) in which an initial dissolution rate (IDR_5_) of 7.79% was observed after 5 min with a dissolution efficiency (%DE_60_) value of 42.48 after 60 min (Table 3).

**Table 2:** Dissolution parameters of pure MA and its SDs prepared using different carriers at drug-carrier ratio of 1: 5.

System	IRD (%)	DE_60_ (%)	RDR_60_ (%)
Pure MA	8.91	7.51	-
MA-GLU (1: 5)	6.12	10.16	1.98
MA-PF (1: 5)	30.59	58.74	8.84
MA-PEG 4000 (1: 5)	59.88	61.18	8.44
MA-EPO (1: 5)	51.69	46.40	6.30

IRD (%): Initial dissolution rate (after 15 min)

DE_60_ (%): Dissolution efficiency after 60 min

RDR_60_(%): Relative dissolution rate after 60 min

**Table 3:** Dissolution parameters of pure FFA and its SDs prepared using different carriers at drug-carrier ratio of 1: 5.

System	IRD (%)	DE_60_ (%)	RDR_60_ (%)
Pure MA	39.46	42.48	-
MA-GLU (1: 5)	25.20	34.95	0.95
MA-PF (1: 5)	19.30	44.98	1.21
MA-PEG 4000 (1: 5)	60.15	59.40	1.26
MA-EPO (1: 5)	23.19	22.48	0.51

IRD (%): Initial dissolution rate (after 15 min)

DE_60_ (%): Dissolution efficiency after 60 min

RDR_60_(%): Relative dissolution rate after 60 min

The dispersion of FFA in the polymeric matrix of GLU caused enhanced initial drug dissolution (IDR was 9.21%) but did not result in enhancing its overall dissolution rate. MA-GLU SD also exhibited a slow dissolution rate with initial burst dissolution of 3.46% (IDR) and DE% of 10.16. The slower dissolution rates of both FFA and MA in their GLU binary systems might be due to the partial hydrophobic nature of GLU whose HLB value is reported as 13.0 [46].

Unfortunately, the SD of FFA acid and MA in EPO matrix showed enhanced initial dissolution (14.4% and 31.83% after 5 min for FFA and MA, respectively) but a slow dissolution rate was depicted during the whole dissolution period (only 23% was dissolved within 60 min). This might be due to a fact that EPO is swellable and permeable above this pH but poorly soluble above this pH [47].

The drugs dissolution rates showed a noticeable enhancement in case of SD in PL and PEG 4000 binary systems. The drug DE% values calculated were 44.89% and 59.40% in case of FFA SD in PL and PEG matrices, respectively. Also, the MA exhibited DE% 58.74 and 61.18 in its-binary systems in PL and PEG matrices, respectively. However, slow initial dissolution rate (IDR) of both FFA and MA were observed from their binary systems with PL. The increase in the dissolution rate of FFA in its binary systems in the matrices of PL and PEG 4000 could be attributed on the basis of the crystallization of drug particles simultaneously in very minute crystals surrounded by water-soluble molecules of the carrier, as revealed by DSC and PXRD data. This results in an increase in the specific surface area subjected to the dissolution medium which in turn enhances drug dissolution rate [45, 48].

Moreover, the hydrophilic SD carrier surrounding the drug particles may reduce aggregation and agglomeration of these particles, resulting in their rapid contact with dissolution medium with wetting of their surfaces, and so increase its dissolution rate [49].

### *In vivo* anti-inflammatory studies

**Fig 13:**
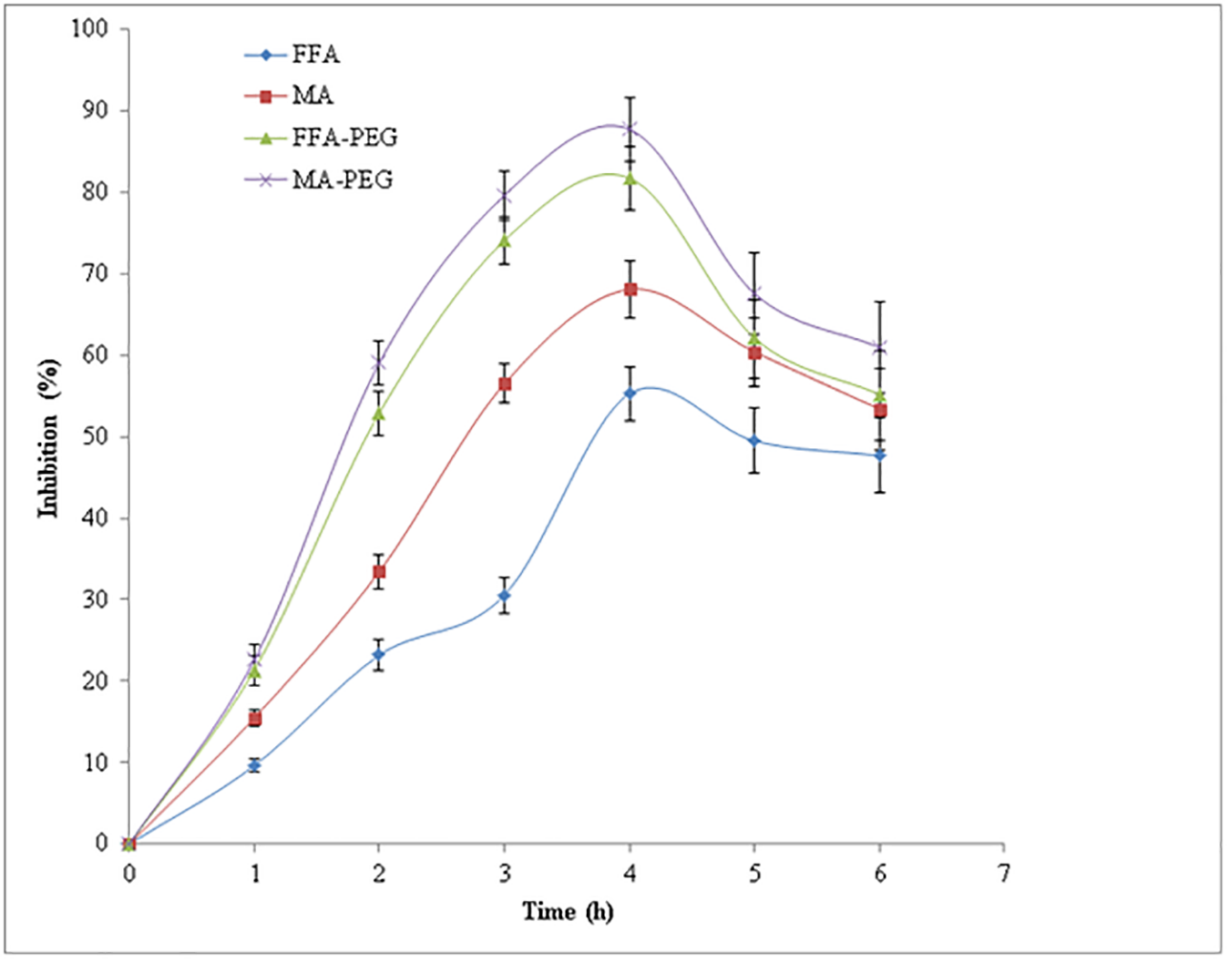
*In vivo* anti-inflammatory effects of MA suspension, FFA suspension, MA-PEG SD and FFA-PEG SD in male Wistar rats.

*In vivo* anti-inflammatory effects of MA/PEG and FFA/PEG SDs after oral administration were compared with those of MA suspension and FFA suspension. The results of *in vivo* anti-inflammatory studies in male Wistar rats are shown in Fig 13. The anti-inflammatory effects of MA suspension, FFA suspension, MA/PEG SD and FFA/PEG SD were found to be increased with increase in time for up to 4 h. However, after 4 h of oral administration, these effects were started to decrease slightly (Fig 13). The % inhibition value after 4 h of oral administration was obtained as 87.74% for MA/PEG SD in comparison with 68.09% for MA suspension. This value of MA/PEG SD was statistically significant in comparison with MA suspension (P < 0.05). The % inhibition value after 4 h of oral administration was obtained as 81.76% for FFA/PEG SD in comparison with only 55.27% for FFA suspension which was statistically highly significant in comparison with FFA suspension (P < 0.05). Overall, *in vivo* anti-inflammatory effects of MA/PEG SD and FFA/PEG SD were highly significant in comparison with their suspension formulations. The enhanced anti-inflammatory effects of MD/PEG SD and FFA/PEG SD were possible due to the rapid absorption of MA and FFA from their SDs due to the presence of solubilizer such as PEG 4000 in comparison with their suspension formulations. The anti-inflammatory effects of MA/PEG SD and FFA/PEG SD were possible due to inhibition of prostaglandins and proinflammatory cytokines inductions.

## Conclusions

In this study, various SDs of two anti-inflammatory drugs MA and FFA were developed using different carriers including PL, EPO, GLU and PEG 400 by MW technology. Prepared SDs of MA and FFA were characterized physicochemically and evaluated for *in vitro* drug release studies. Physicochemical characterization of prepared SDs indicated that SDs of MA and FFA were developed successfully. *In vitro* drug release studies showed that SDs of MA and FFA prepared using PEG 4000 as carrier presented maximum drug release profile in comparison with pure MA, pure FFA and other SDs. Based on the best physicochemical parameters and maximum drug release profile, MA/PEG SD and FFA/PEG SD were selected for *in vivo* anti-inflammatory studies in male Wistar rats. *In vivo* anti-inflammatory studies in male Wistar rats showed that MA and FFA in their SDs were highly efficacious than their suspension formulations. MW SDs of MA as well as FFA were proved to be an excellent carriers for their oral administration. Overall, the results of this study showed that the MW SDs could be successfully used in the enhancement of *in vitro* dissolution rate and anti-inflammatory efficacy of poorly soluble drugs such as MA and FFA.
